# The Chinese herbal medicine FTZ attenuates insulin resistance via IRS1 and PI3K in vitro and in rats with metabolic syndrome

**DOI:** 10.1186/1479-5876-12-47

**Published:** 2014-02-20

**Authors:** Xuguang Hu, Man Wang, Weijian Bei, Zongyu Han, Jiao Guo

**Affiliations:** 1 Key Unit of Modulating Liver to Treat Hyperlipemia SATCM (State Administration of Traditional Chinese Medicine), Level 3 Lab of Lipid Metabolism SATCM, Guangdong TCM key laboratory for metabolic diseases, Guangdong Pharmaceutical University, Guangzhou Higher Education Mega Centre, Guangzhou 510006, PR China

**Keywords:** Metabolic syndrome, Insulin resistance, Fu Fang Zhen Zhu Tiao Zhi formula (FTZ)

## Abstract

**Background:**

Insulin resistance plays an important role in the development of metabolic syndrome (MS). Fu Fang Zhen Zhu Tiao Zhi formula (FTZ), a Chinese medicinal decoction, has been used to relieve hyperlipidemia, atherosclerosis and other symptoms associated with metabolic disorders in the clinic.

**Methods:**

To evaluate the effect of FTZ on insulin resistance, HepG2 cells were induced with high insulin as a model of insulin resistance and treated with FTZ at one of three dosages. Next, the levels of glucose content, insulin receptor substrate1 (IRS1) protein expression and phosphatidylinositol 3-kinase (PI3K) subunit p85 mRNA expression were measured. Alternatively, MS was induced in rats via gavage feeding of a high-fat diet for four consecutive weeks followed by administration of FTZ for eight consecutive weeks. Body weight and the plasma levels of lipids, insulin and glucose were evaluated. Finally, the expression of PI3K p85 mRNA in adipose tissue of rats was measured.

**Results:**

Our results revealed that FTZ attenuated glucose content and up-regulated the expression of PI3K p85 mRNA and IRS1 protein in insulin-resistant HepG2 cells in vitro. Moreover, FTZ reduced body weight and the plasma concentrations of triacylglycerol, cholesterol, fasting glucose and insulin in insulin resistant MS rats. FTZ also elevated the expression of PI3K p85 mRNA in the adipose tissues of MS rats.

**Conclusion:**

FTZ attenuated MS symptoms by decreasing the plasma levels of glucose and lipids. The underlying mechanism was attenuation of the reduced expression of PI3K p85 mRNA and IRS1 protein in both insulin-resistant HepG_2_ cells and MS rats.

## Background

Reaven noted that dyslipidemia, hypertension and hypertriglyceridemia often occurred together [1]. Consequently, this author proposed the concept of X Syndrome, regarding insulin resistance as the primary characteristic of X Syndrome. Based on the association of X syndrome with a variety of metabolic diseases, Zimmet et al. defined its symptoms as metabolic syndrome (MS). MS is related to excessive energy intake, a sedentary lifestyle, and multiple genetic factors [2]. Many studies have demonstrated that patients with MS displayed an increased risk of developing diabetes, cardiovascular disease and other diseases. Insulin resistance (IR) has been suggested as the common pathophysiological basis of MS. Because there is no eutherapeutic Western medicine to treat MS, searching for an effective therapeutic among the traditional Chinese medicines (TCMs) or botanicals is urgently required.

Fu Fang Zhen Zhu Tiao Zhi formula (FTZ), for which a patent has been issued, is composed of eight medical herbs and has been proven clinically effective for the treatment of dyslipidemia [3,4]. Our previous study demonstrated that FTZ could reduce serum levels of total cholesterol (TC), triglycerides (TG) and low-density lipoprotein cholesterol (LDL-C) and increase the serum level of high-density lipoprotein cholesterol (HDL-C) in hyperlipidemic patients [5,6]. FTZ could reduce the viscosity of plasma and whole blood in hyperlipidemic patients [7]. In addition, our previous studies also demonstrated the antioxidant properties of FTZ, which reduced the oxidation of LDL-C [8]. FTZ also could improve serum lipid profile in high lipid diet induced hyperlipidemic rats by regulating the HMG-CoA reductase and CYP7A1 [9].

MS includes the symptoms dyslipidemia, hypertension and hypertriglyceridemia and is typically characterized by insulin resistance. Therefore, insulin resistance is suggested to be a new drug target for treatment of MS or dyslipidemia. Nevertheless, the effect and mechanisms of FTZ on MS and insulin resistance remain unclear.

To elucidate the effect of FTZ on insulin resistance and its mechanism of action, HepG2 cells with insulin resistance and MS rats were used, and the effect of FTZ on insulin-resistant HepG2 cells and MS rats was measured. Moreover, the resistance index, the gene expression level of PI3K and the protein expression level of IRS1 were also investigated.

## Methods

### Materials

HepG2 cells were purchased from the American Type Culture Collection (ATCC) bioresource center (Manassas, VA, USA). Dulbecco’s modified Eagle’s medium (DMEM) and fetal bovine serum (FBS) were purchased from Invitrogen (Carlsbad, CA, USA). IRS1 and GADPH antibodies were from Abcam Inc. (Cambridge, MA, USA). Insulin and rosiglitazone (RGS) were purchased from Sigma (St. Louis, MO, USA). All other reagents were analytical grade.

### Preparation of FTZ extract

Herbs in FTZ [composed of *Ligustrum lucidum* W.T.Aiton, fructus; *Atractylodes macrocephala* Koidz., rhizoma; *Salvia miltiorhiza* Bunge, radix; *Coptis chinensis* Franch., rhizoma; *Panax notoginseng* (Burk.)F.H.Chen, radix; *Eucommia ulmoides* Oliv., cortex; *Cirsium japonicum* (Thunb.) Fisch. ex DC., radix; *Citrus medica var. sarcodactylus (Siebold ex Hoola van Nooten)* Swingle, fructus] were provided by Zhixin Chinese Herbal Medicine Co.,Ltd. (Guangzhou, China) and authenticated by Professor Shuyan Li, pharmacognosist of School of Chinese Medicinal Sciences, Guangdong Pharmaceutical University. All of the raw materials in FTZ were examined according to the quality control criteria of Chinese Pharmacopeia 2010 and were controlled as previously reported [10]. The FTZ extract was prepared via alcohol and water extraction of eight herbs according to the protocol (see Supporting Material for the protocol of FTZ preparation). FTZ was obtained from the Institute of Materia Medica, Guangdong Pharmaceutical University. The voucher specimens were GDPUZYY 20110901-8(see Supporting Material-Voucher specimen). Quality analysis of the FTZ extract was performed via HPLC fingerprinting, which was obtained using a HPLC unit (Waters, USA) with an Agilent HC-C18 column (4.6 mm × 250 mm, 5 μm). All assigned peaks were identified by performing a co-injection test with authentic samples and comparative analysis of the UV spectral data (see Supporting Material-HPLC conditions) [9].

### Cell culture

The human hepatocellular carcinoma cell line HepG2 was purchased from ATCC. Cells were cultured in DMEM supplemented with 10% heat-inactivated fetal FBS at 37°C in a 5% CO_2_ atmosphere. In all experiments, cells grew to 80-90% confluence.

### Induction of insulin-resistance in HepG2 cells and glucose uptake experiments

Insulin resistance was induced in HepG2 cells as previously described [11-13]. In brief, HepG2 cells were seeded on 24-well plates at 2 × 10^5^ cells/well, incubated for 24 h to reach maximal confluence and serum-starved for another 24 h. The cells were then incubated for 36 h in serum-free DMEM containing 25 mmol/l d-glucose, 10^-6 ^mol/l insulin in the absence or presence of 1, 25 and 100 μg/ml FTZ or 10 μmol/l RGS. FTZ administration at 100, 25 and 1 μg/ml were defined as high, medium and low dosages, respectively. Next, cells were washed twice with PBS. The cells were then incubated for 24 h in serum-free DMEM without phenol red. The glucose content was quantified using a GOD-POD kit, measuring optical absorbance at 505 nm.

### Western blot analysis

Cells were washed with ice-cold PBS and lysed with lysis buffer (50 mmol/l Tris HCl, 1% Triton X-100, 0.5% sodium deoxycholate, 150 mmol/l NaCl, 1 mmol/l EDTA, 1 mmol/l PMSF, 1 mmol/l sodium orthovanadate, 1 mmol/l NaF and 0.2% protease inhibitor cocktail; pH7.2). For western blotting, protein samples (20 μg) of high insulin-induced insulin-resistant HepG2 cells were separated via 10% sodium dodecyl sulfate-polyacrylamide gel electrophoresis (SDS-PAGE). The proteins were transferred to a PVDF membrane and incubated with primary antibody (anti-IRS1, or anti-GAPDH), followed by secondary antibody (horseradish peroxidase-conjugated anti-rabbit IgG). Intensity of the immunoblot signal was assayed using Western Bright™ ECL spray and analyzed quantitatively using GeneTools software from Syngene (Syngene, Cambridge, UK).

### RNA isolation and quantitative real-time PCR

Total RNA was isolated from HepG2 cells using TRIzol® reagent (Invitrogen, Carlsbad, CA, USA) dissolved in DEPC-treated water according to the manufacturer’s instructions. RNA (2 μg) was reverse-transcribed to cDNA using oligo(dT) primers and moloney murine leukemia virus reverse transcriptase (RT) in a final volume of 20 μl under the conditions recommended by the supplier (Invitrogen). The resulting cDNA was amplified using primers specific for PI3K or β-actin in a total volume of 10 μl. Quantitative real-time PCR was performed using a Roche LightCycler 480 PCR system using SYBR green fluorescence. The expression level of PI3K was normalized to that of β-actin, which was used as a specific endogenous control. Primer sequences used were as follows:

PI3K forward 5′-TGG ACG GCG AAG TAA AGC ATT-3′, reverse 5′-AGT GTG ACA TTG AGG GAG TCG-3′; β-actin forward 5′-AGC CAT GTA CGT AGC CAT CC-3′, reverse 5′- ACC CTC ATA GAT GGG CAC AG-3′.

### Induction of metabolic syndrome in rats and experiment protocol

Male Sprague Dawley rats (180-220 g) were obtained from the Laboratory Animal Center of Guangzhou University of Chinese Medicine (Guangzhou, Republic of China). Rats were housed in a climate-controlled environment (25±1°C at 50% relative humidity) with 12-h light/12-h dark cycles. Water was available *ad libitum*. The model rats of metabolic syndrome (MS) were induced by fed a high-fat diet (HFD) consisting of 40% (wt/wt) fat for 12 consecutive weeks, while control rats were fed normal chow. Then the MS rats were randomly divided into 5 groups and treated with distilled water (Group 1), FTZ -H (Group 2), FTZ -M (Group 3), FTZ -L (Group 4) and aspirin (200 mg/kg) (Group 5), respectively. FTZ with 6 g/kg, 3 g/kg and 1.5 g/kg were defined as high, medium and low dosages, respectively. From the fifth week to twelfth week, rats of FTZ groups were treated by FTZ for 8 consecutive weeks. Then the rats were used to measure body weight (BW) and serum levels of total cholesterol (TC), triglycerides (TG) and high-density lipoprotein cholesterol (HDL-C). Subsequently, all the rats were sacrificed and their epididymis and kidney fat were excised for PI3K mRNA assay. The animal care and study protocols were maintained in accordance with the provisions and general recommendation of the Chinese Regulations for the Administration of Affairs Concerning Experimental Animals at Guangdong Pharmaceutical University.

### Determination of body weight and biochemical indexes

During the animal experiments, body weight (BW) was recorded at 0, 4, 8 and 12 weeks. Serum levels of total cholesterol (TC), triglycerides (TG) and high-density lipoprotein cholesterol (HDL-C) were analyzed using a blood chemistry analyzer (Hitachi 7040).

### Assessment of insulin resistance(IR)

Fasting plasma glucose level was measured via glucose oxidase method. Fasting plasma insulin was measured using radioimmunoassay method. IR was assessed according to the homeostasis model assessment index(HOMA-IR) calculated using the following formula: (fasting plasma insulin level[μU/ml] × fasting plasma glucose [mmol/l]) /22.5 [14].

### RNA isolation and RT-PCR

Total RNA was isolated from adipose tissue using TRIzol® reagent (Invitrogen, Carlsbad, CA, USA) dissolved in DEPC-treated water according to the manufacturer’s instructions. RNA (2 μg) was-reverse transcribed to cDNA using oligo (dT) primers and moloney murine leukemia virus reverse transcriptase (RT) in a final volume of 20 μl under the conditions recommended by the supplier (Invitrogen). For polymerase chain reaction (PCR) amplification of PI3K and β-actin, 1 μl of cDNA template and the following specific primers were used: PI-3K p85 forward 5’-GAA GGC AAC GAG AAG GA-3’, reverse 5’-CAC AAG TGT CAG CCA CAT-3’ (213bp); β-actin forward 5’-AGA TCT GGC ACC ACA CCT TCT AC-3’, reverse 5’-TCA GGA TCT TCA TGA GGT AGT CT-3’ (388bp). The reaction cycle conditions were: denaturation at 90°C for 50 s, annealing at 56.1°C for 50 s and extension at 72°C for 50 s. The PCR products were resolved using a 2% agarose gel and visualized with ethidium bromide staining. The expression level of PI3K was normalized to that of β-actin, which was used as a specific endogenous control.

### Statistics

Statistical analyses were conducted using SPSS16.0 software. All results are presented as the mean ± standard deviation (SD). Statistical analysis was performed via analysis of variance (one-way ANOVA) followed by the Student-Newman-Keuls test for significance. Differences were considered statistically significant at P< 0.05.

## Results

### Effect of FTZ on glucose content in insulin-resistant HepG2 cells

**Figure 1:**
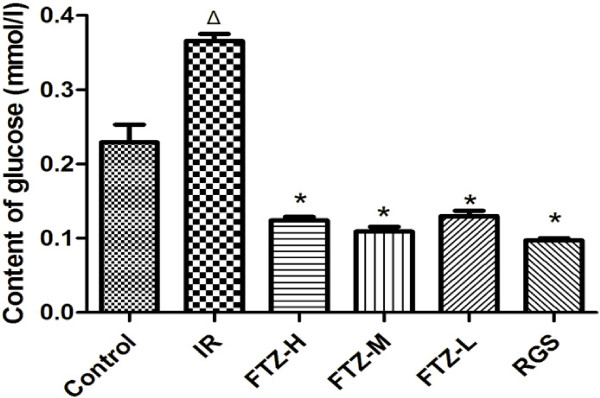
**Effect of FTZ on glucose content in HepG2 cells.** HepG2 cells (2 × 10^5 ^cells/well) were incubated for 36 h in serum-free DMEM containing 10^-6^mol/l insulin in the absence or presence of FTZ or RGS. The content of glucose was quantified using a GOD-POD kit. Δ*P*<0.05 compared to the control cells; *P <0.05 compared to the IR cells.

The glucose content in insulin-resistant HepG2 cells in culture medium significantly increased compared to that of control cells. After treatment with FTZ (1, 25 and 100 μg/ml), glucose content in the culture medium significantly decreased compared to that of IR cells (P<0.05). RGS (10 μmol/l), used as a positive control drug, was also able to increase glucose content in the culture medium (Figure 1).

### Effect of FTZ on PI-3K p85 mRNA expression in insulin-resistant HepG2 cells

**Figure 2:**
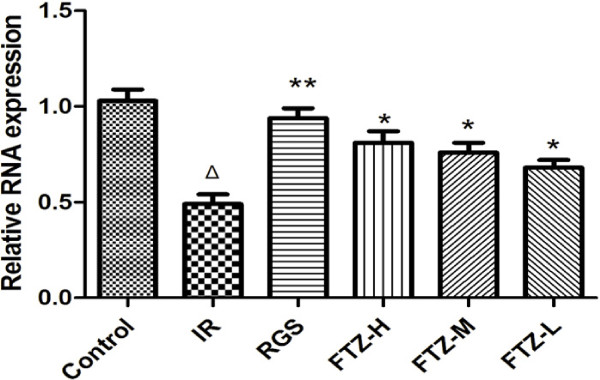
**Effect of FTZ on PI-3K p85 mRNA expression.** The expression of PI-3K p85 mRNA was detected via quantitative real-time PCR as described in the text. Δ*P* <0.05 compared to the control cells; *P <0.05, **P <0.01 compared to the IR cells.

To evaluate the effect of FTZ on PI-3K p85 mRNA expression, total RNA was extracted from HepG2 cells, and real-time PCR was performed. As shown in Figure 2, PI-3K p85 mRNA expression in HepG2 cells with IR was decreased compared to control cells (P<0.05 or P<0.01). After treatment with FTZ, PI-3K p85 mRNA expression significantly increased compared to IR cells (P<0.05). These results suggest that FTZ induces an insulin sensitizing effect on IR cells through the up-regulation of PI-3K p85 mRNA expression in HepG2 cells (Figure 2).

### Effect of FTZ on IRS1 protein expression in HepG2 cells with insulin resistance

**Figure 3:**
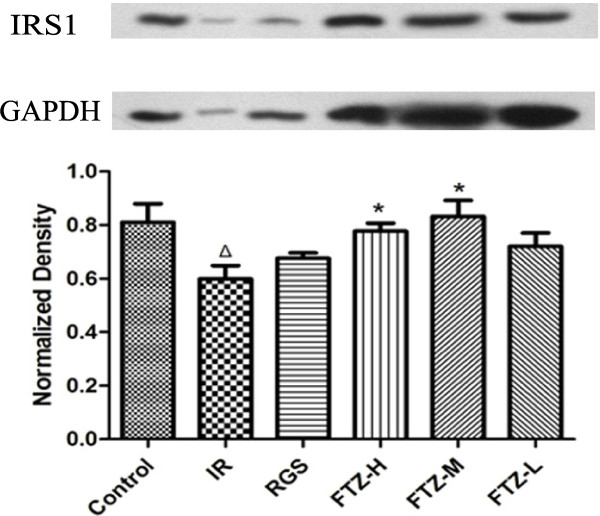
**Effect of FTZ on IRS1 protein expression.** The protein expression of IRS1 was detected via western blotting as described in the text. The figure represents one of three experiments with similar results. Lane1, control; Lane2, IR (FTZ 0 μg/ml); Lane3, RGS 10 μmol/l; Lane4, FTZ 100 μg/ml; Lane5, FTZ 25 μg/ml; Lane 6, FTZ 1 μg/ml. Δ*P*<0.05 compared to the control cells; *P< 0.05 compared to the IR cells.

To elucidate the effects of FTZ, western blot analysis was used to measure IRS1 protein expression in HepG2 cells. As shown in Figure 3, IRS1 protein expression was significantly reduced compared to control cells (P<0.05). After treatment with FTZ, IRS1 protein expression was significantly increased compared to IR cells (P<0.05) (Figure 3).

### Effect of FTZ on body weight of MS rats

**Figure 4:**
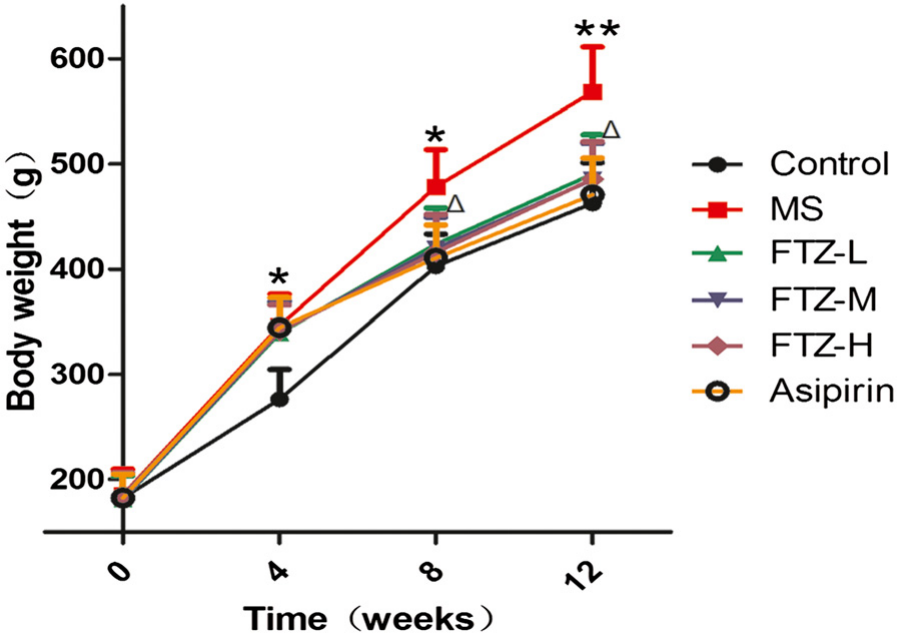
**Effect of FTZ on body weight of MS rats (g). **The body weight was recorded at 0, 4, 8, and 12 weeks. Aspirin (10 ml/kg/day), used as a positive control drug, was able to decrease body weight of MS rats. Data are mean ± SE (*n* = 10 for each group); *P<0.05, **P<0.01 compared to the control rats; Δ*P*<0.05 compared to the MS rats.

After the rats were fed a high-fat diet for 12 continuous weeks, our results indicated that the body weight of MS rats was significantly higher than that of control rats (P <0.05, P <0.01). After high, medium or low dosage FTZ treatment for 4 consecutive weeks (equivalent to the rats fed a high-fat diet for 8 weeks), the body weight of the rats in the FTZ groups was significantly reduced compared to that of MS rats. After FTZ treatment for 8 consecutive weeks (equivalent to the rats fed a high-fat diet for 12 weeks), the high, medium and low dosage FTZ-treated groups all possessed body weights which were significantly reduced compared to the MS rats. (P < 0.05, P < 0.01) (Figure 4).

### Effect of FTZ on blood biochemical index of MS rats

**Figure 5:**
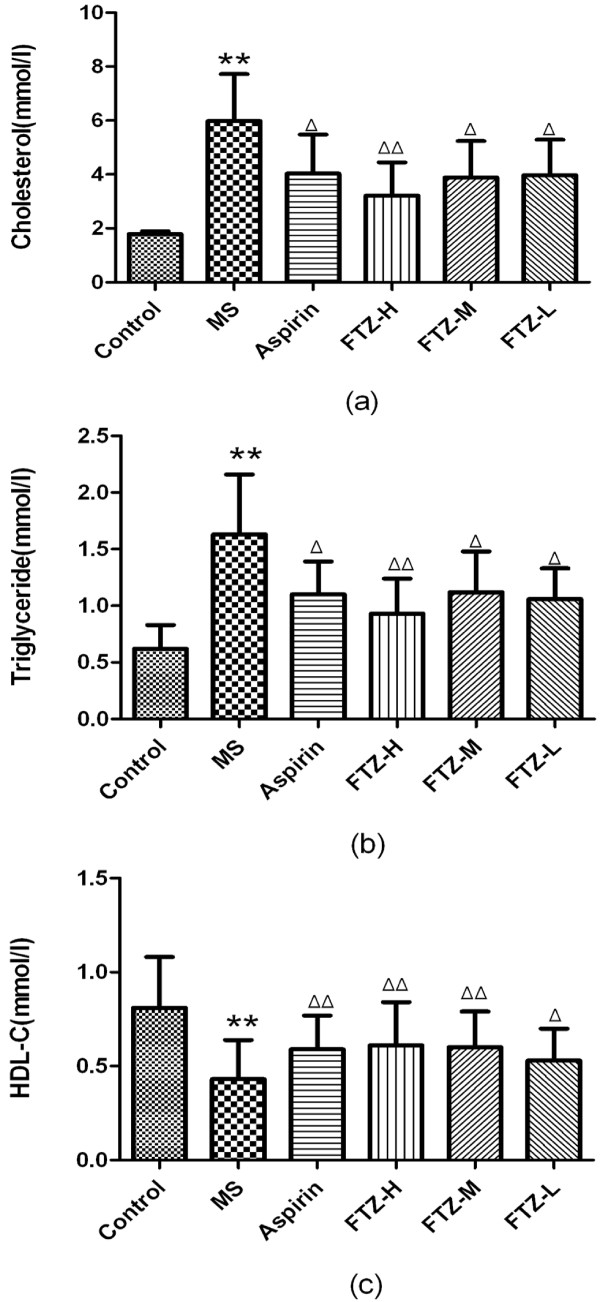
**Blood biochemical indexes (cholesterol, triglyceride and HDL-C) of MS rats.** Blood samples were collected from the orbital sinus, and cholesterol **(a)**, triglyceride **(b)**, HDL-C **(c)** levels were measured following administration of FTZ at 6 g/kg/day, 3 g/kg/day or 1.5 g/kg/day, aspirin (AS) at 10 ml/kg/day or saline (control rats) for 8 consecutive weeks in high-fat diet-treated rats. *P<0.05, **P<0.01 compared to the control rats; Δ*P*<0.05, ΔΔ*P*<0.01 compared to the MS rats.

Compared to the control rats, the levels of serum TG and TC in MS rats were significantly increased (P<0.05 or P<0.01), while the level of serum HDL-C was significantly reduced (P<0.05 or P<0.01). After treatment with FTZ, the serum levels of TG and TC in MS rats were significantly reduced (P<0.05 or P<0.01), and the serum level of HDL-C was significantly increased (P<0.05 or P<0.01). These results revealed that FTZ could alter the serum levels of lipids in MS rats (Figure 5a-c).

### Effect of FTZ on fasting glucose, insulin and HOMA-IR index in MS rats

**Figure 6:**
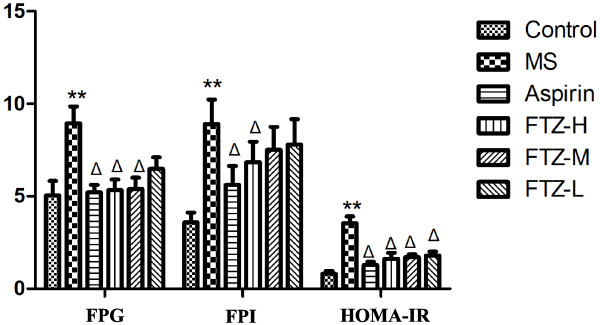
**Other blood biochemical indexes (fasting glucose, insulin and HOMA-IR index) of MS rats.** Fasting plasma glucose (FPG) level was measured via the glucose oxidase method. Fasting plasma insulin (FPI) in rats was measured using a radioimmunoassay method. To quantify the insulin resistance index, the following formula was used: HOMA-IR = {(FPG*FPI)/22.5}. **P<0.01 compared to the control rats; Δ*P*<0.05 compared to the MS rats.

Compared to the control rats, levels of fasting glucose and insulin, as well as the HOMA-IR index, were significantly higher in MS rats (P<0.05 or P<0.01). After treatment with the high dosage of FTZ or aspirin, the levels of fasting glucose and insulin and HOMA-IR index were all significantly reduced compared to the MS rats (P<0.05 or P<0.01). After treatment with the medium dosage of FTZ, the fasting glucose level and the HOMA-IR index were significantly reduced compared to the MS rats (P<0.05 or P<0.01). After treatment with the low dosage of FTZ, the HOMA-IR index was significantly reduced compared to the MS rats (P<0.05 or P<0.01) (Figure 6).

### Effect of FTZ on PI-3K p85 mRNA expression in adipose tissue

**Figure 7:**
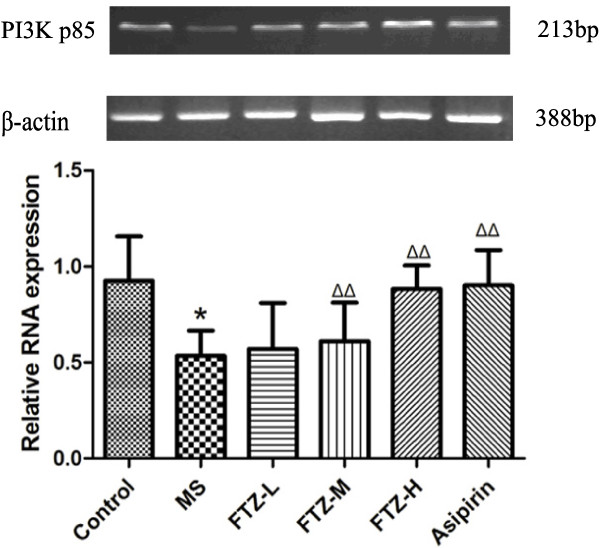
**Effect of FTZ on PI-3K p85 mRNA expression.** The expression of PI-3K p85 mRNA was detected via RT-PCR as described in the text. *P<0.05 compared to the control rats; Δ*P* <0.05, ΔΔ*P*<0.01 compared to the MS rats.

To evaluate the effect of FTZ on PI-3K p85 mRNA expression, we performed RT-PCR in the adipose tissue of rats. As shown in Figure 7, compared to the control rats, the MS rats produced a lower expression level of PI-3K p85 mRNA (P<0.05 or P<0.01). Administration of either aspirin or FTZ, the expression of PI-3K p85 mRNA was increased compared to the MS rats (Figure 7).

## Discussion

This study revealed that the Chinese herbal formula FTZ could attenuate MS symptoms by decreasing the content of glucose and lipids, and well as the insulin resistance index. Additionally, its effects were possibly mediated through increased expression of PI-3Kp85 mRNA and IRS1 protein in insulin-resistant HepG2 cells and MS rats. Insulin resistance has been suggested as an underlying cause of MS, including hyperglycemia, dyslipidemia and type 2 diabetes mellitus.

In our study, HepG2 cells were used as an insulin resistance model to investigate the effect of FTZ on glucose metabolism and insulin signaling. HepG2 cells express PI-3Kp85 and IRS1 genes, which are involved in the insulin signaling pathway [15,16]. Therefore, these cells have been widely used to analyze glucose metabolism, lipid metabolism, and insulin resistance [17,18].

Defects in the insulin signaling cascade, which lead to impaired glucose utilization, were believed to play a key role in the pathogenesis of insulin resistance [19]. It is conceivable that IRS-1 tyrosine phosphorylation in response to insulin stimulation generally increased the association of IRS-1 with PI 3-kinase, resulting in increased PI 3-kinase activity, which in turn led to activation of serine/threonine kinase protein B (PKB or Akt) and, ultimately, to an enhancement in insulin-stimulated glucose disposal [20]. Our research results revealed that the insulin receptor was impaired, producing an insulin-resistant state in HepG2 cells under high insulin conditions. The expression of the IRS-1 protein and IRS-1-associated PI-3K activity in HepG2 cells were significantly decreased. After treatment with FTZ, the expression of IRS-1 protein and PI-3K mRNA were partially restored. Here, we revealed that the FTZ-mediated recovery of insulin action was related to the improvement of the IRS-1/PI 3-kinase signaling pathway in insulin-resistant HepG2 cells. It appears that a FTZ-mediated improvement in post-receptor insulin signaling may have induced the subsequent increase in insulin sensitivity.

In our study, MS model rats were induced via high-fat diet feeding for 4 weeks. This model exhibited hyperinsulinemia, obesity, decreased insulin sensitivity, dyslipidemia and other features [21]. In our study, the MS rats exhibited increased body weight, levels of serum TG and total cholesterol, fasting glucose and plasma insulin, as well as an increased insulin resistance index. This was consistent with previous studies, such as I-Min Liu et al. [22]. After treatment with FTZ, body weight, levels of serum TG and TC, fasting glucose and plasma insulin and the insulin resistance index were significantly reduced compared to MS rats. FTZ treatment also enhanced the activity of PI3K in adipose tissue compared to MS rats.

Our study suggested that FTZ might ameliorate insulin resistance and treat MS. This effect might be associated with the compounds which it contained. It has been reported that oleanolic acid (OA) in *Ligustrum lucidum* W.T. Aiton decreased serum triglyceride, total cholesterol, LDL and free fatty acids, increased serum HDL and reduced hepatic lipid accumulation. Furthermore, inflammation in db/db mice was improved by OA, as evidenced by decreased levels of IL-1 β, IL-6, and TNF-α in the circulation and in the liver. These results suggested that OA improved hepatic insulin resistance via inhibition of mitochondrial ROS, hypolipidemia and anti-inflammatory effects [23]. Ginsenoside Re in *Panax notoginseng* (Burk.) F.H. Chen reduced insulin resistance via activation of the PPAR-γ pathway by directly increasing the expression of PPAR-γ2 and its responsive genes, adiponectin, IRS-1 and ap2, inhibiting TNF-α production and facilitating the translocation of GLUT4 to promote glucose uptake and disposal in 3T3-L1 adipocytes [24]. Berberine in *Coptis chinensis* Franch. improved insulin-induced tyrosine phosphorylation of IRS-1 and the recruitment of p85 to IRS-1. The ameliorated insulin signal transduction was related to berberine-mediated inhibition of mTOR, which attenuated serine phosphorylation of IRS-1. These results suggested that berberine might ameliorate insulin resistance by modulating key molecules in the insulin signaling pathway, leading to increased glucose uptake in insulin-resistant cells [25]. Therefore, we suspect that these ingredients might explain the role of FTZ in ameliorating insulin resistance.

## Conclusion

In conclusion, our study indicated that FTZ could decrease serum triglyceride, total cholesterol and fasting blood glucose and increase serum HDL-C, thereby reactivating the insulin-stimulated IRS1/PI3K pathway in insulin-resistant HepG2 cells and up-regulating PI3K expression in adipose tissue. Therefore, the beneficial effects of FTZ on insulin resistance suggest that this decoction may be a promising therapeutic for MS and insulin resistance.

## Abbreviations

FTZ: Fu Fang Zhen Zhu Tiao Zhi formula; MS: Metabolic syndrome; IR: Insulin resistance; IRS1: Insulin receptor substrate-1; PI3K: Phosphatidylinositol 3-kinase; TG: Triglyceride; TC: Total cholesterol; HDL-C: HDL-cholesterol; FPG: Fasting plasma glucose; FPI: Fasting plasma insulin; HOMA-IR: Homeostasis model assessment- insulin resistance index.

## Competing interests

The author(s) declare that they have no competing interests.

## Authors’ contributions

Dr. J.Guo and Xuguang Hu designed the study. Man Wang carried out experiments. Bei WJ and Wang LY, participated in the design of study, interpretation of results, and drafted the manuscript. Mr. Shuyan Li, Zongyu Han, Xiuteng Zhou, Le Cao, Hu Yinming, Ms. Wei He, Junhui Peng and Duosheng Luo have took part in the research projects. All authors have read and approved the final manuscript.

## References

[B1] Reaven GM Role of insulin resistance in human disease Diabetes 1988 37 1595 1607 10.2337/diab.37.12.1595 3056758

[B2] Zimmet PZ McCarty DJ The global epidemiology of non-insulin-dependent diabetes mellitus and the metabolic syndrome J Diabetes Complications 1997 11 60 68 10.1016/S1056-8727(96)00090-6 9101389

[B3] Guo J A drug for the treatment of hyperlipidemia[P] 2004 China 0410051250.4

[B4] Cao Y Bei W Hu Y Cao L Huang L Wang L Luo D Chen Y Yao X He W Liu X Guo J Hypocholesterolemia of Rhizoma Coptidis alkaloids is related to the bile acid by up-regulated CYP7A1 in hyperlipidemic rats Phytomedicine 2012 19 686 692 10.1016/j.phymed.2012.03.011 22554715

[B5] Tong G Jiao G Wei H The clinical observation of Fufang Zhenshu Tiaozhi on hyperlipemia Journal of Shandong University of TCM 2006 30 204 206

[B6] Guo J Bei WJ Tang CP The effect of Fufang Zhenshu Tiaozhi extract on hepatic lipase in diet-induced hyperlipidemic rats Zhong Yao Cai 2009 32 582 585

[B7] Tong G Jiao G Yingyu L The effect of Fufang Zhenshu Tiaozhi on the viscosity of plasma and whole blood in hyperlipidemic patients Journal of Practical traditional Chinese medicine 2006 22 10 608 609

[B8] Wu HY Bei WJ Guo J Chinese herbal medicine for the treatment of dyslipidemia J Geriatr Cardiol 2009 6 119 125

[B9] Guo J Bei WJ Hu YM Tang CP He W Liu XB Huang LH Cao Y Hu XG Zhong XL Cao L A new TCM formula FTZ lowers serum cholesterol by regulating HMG-CoA reductase and CYP7A1 in hyperlipidemic rats J Ethnopharmacol 2011 135 299 307 10.1016/j.jep.2011.03.012 21396994

[B10] Xunlong Z Jiao G Laiyou W Analysis of the constituents of the prototype and metabolite constituents in rat serum after oral administration of Fu Fang Zhen Zhu Tiao Zhi capsule by UPLC-Q-TOF MS/MS Chromatographia 2012 75 111 129 10.1007/s10337-011-2164-6 22307991 PMC3264872

[B11] Zhou L Meng Q Qian T Yang Z Ginkgo biloba extract enhances glucose tolerance in hyperinsulinism-induced hepatic cells J Nat Med 2011 65 50 56 10.1007/s11418-010-0456-z 20814756

[B12] Zhang WY Scopoletin improves insulin resistance by scopoletin in high-glucose-induced, insulin-resistant HepG2 cells Horm Metab Res 2010 42 930 935 10.1055/s-0030-1265219 20886413

[B13] Renstrfm F Insulin resistance induced by high glucose and high insulin precedes insulin receptor substrate 1 protein depletion in human adipocytes Metabolism Clinical and Experimental 2007 56 190 198 10.1016/j.metabol.2006.09.012 17224332

[B14] Salgado AL Carvalho L Oliveira AC Santos VN Vieira JG Parise ER Insulin resistance index (HOMA-IR) in the differentiation of patients with non-alcoholic fatty liver disease and healthy individuals Arq Gastroenterol 2010 47 2 165 169 10.1590/S0004-28032010000200009 20721461

[B15] Nakamaru K Matsumoto K Taguchi T Suefuji M Murata Y Igata M Kawashima J Kondo T Motoshima H Tsuruzoe K Miyamura N Toyonaga T Araki E AICAR An activator of AMP-activated protein kinase, down-regulates the insulin receptor expression in HepG2 cells Biochem Biophys Res Commun 2005 328 449 454 10.1016/j.bbrc.2005.01.004 15694368

[B16] Woods A Zzout-Marniche D Foretz M Stein SC Lemarchand P Ferre P Foufelle F Carling D Characterization of the role of AMP-activated protein kinase in the regulation of glucose activated gene expression using constitutively active and dominant negative forms of the kinase Mol Cell Biol 2000 20 6704 6711 10.1128/MCB.20.18.6704-6711.2000 10958668 PMC86183

[B17] Zhou L Sell H Eckardt K Yang Z Eckel J Conditioned medium obtained from in vitro differentiated adipocytes and resistin induce insulin resistance in human hepatocytes FEBSLett 2007 581 4303 4308 10.1016/j.febslet.2007.07.076 17716671

[B18] Zang M Zuccollo A Hou X Nagata D Walsh K Herscovitz H Brecher P Ruderman NB Cohen RA AMP-activated protein kinase is required for the lipid-lowering effect of metformin in insulin-resistant human HepG2 cells J Biol Chem 2004 279 47898 47905 10.1074/jbc.M408149200 15371448

[B19] Shulman GI Cellular mechanisms of insulin resistance J Clin Invest 2000 106 171 176 10.1172/JCI10583 10903330 PMC314317

[B20] Carvalho E Rondinone C Smith U Insulin resistance in fat cells from obese Zucker rats-evidence for an impaired activation and translocation of protein kinase B and glucose transporter 4 Mol Cell Biochem 2000 206 7 16 10.1023/A:1007009723616 10839189

[B21] Stanhope KL Havel PJ Fructose consumption: potential mechanisms for its effects to increase visceral adiposity and induce dyslipidemia and insulin resistance Curr Opin Lipidol 2008 19 16 24 10.1097/MOL.0b013e3282f2b24a 18196982 PMC4151171

[B22] Liu I-M Tzeng T-F Liou S-S A Chinese herbal decoction, Dang Gui Bu Xue Tang, prepared from Radix Astragali and Radix Angelicae sinensis, Ameliorates insulin resistance induced by a high-fructose diet in rats Evid Based Complement Alternat Med 2011 2011 1 11 10.1093/ecam/nep004 PMC3095507 19233878

[B23] Wang X Liu R Zhang W Zhang X Liao N Wang Z Li W Qin X Hai C Oleanolic acid improves hepatic insulin resistance via antioxidant, hypolipidemic and anti-inflammatory effects Mol Cell Endocrinol 2013 376 70 80 10.1016/j.mce.2013.06.014 23791844

[B24] Yang G Min-fei Y Ya-ping S Hui-min J Xiao-juan Y Yin-jing Y Hai-long Z Ginsenoside Re reduces insulin resistance through activation of PPAR-γ pathway and inhibition of TNF-α production J Ethnopharmacol 2013 147 509 516 10.1016/j.jep.2013.03.057 23545455

[B25] Li-Zhong L Stanley C.K C Lin-Lin L Stanley K.S H Hong-Xi X Julian C.N C Peter C.Y T Berberine modulates insulin signaling transduction in insulin-resistant cells Mol Cell Endocrinol 2010 317 148 153 10.1016/j.mce.2009.12.027 20036710

